# Editorial

**Published:** 1875-08

**Authors:** 


### Editorial.

***Valedictory.***

With the completion of the present number the Junior Editor retires from the management of, and severs his connection with, the CHICAGO MEDICAL JOURNAL.

The necessity for taking this step has become more and more apparent for many months, during which the increasing demands upon his time, made by duties more strictly personal and professional, have left too little of it for the satisfactory performance of the duties of a journalist.

The preparation and delivery of a course of lectures on Neurology, at Rush Medical College, extending through nine months in each year, the Clinics during the entire year, and the charge of the Department of Nervous and Mental Diseases, at St. Joseph's Hospital, together with the special character of his private practice, demand his exclusive attention to the field of neurological study, and forbid indulgence in the desultory work of editing a journal so general in its scope as this.

But the feeling of relief which comes when the editorial pencil is dropped at last from the wearied fingers, is mingled with regret at the discontinuance of associations maintained harmoniously during nearly eight years—associations which will always be recalled with gratification as unmarked by a single unpleasant incident, however trivial.

His thanks are due, and are most heartily rendered, to all his associates in the work, to Editor and to Publishers, who have so kindly and cordially co-operated with him to make that work successful, and also to the members of the profession at large, who have contributed their talents and their labor to make success secure. To these, and to all, who have endorsed his course in the conduct of the JOURNAL, by the evidences of their approval, with the best wishes for its and their continued success and prosperity, he bids a kind farewell. H.

***Personals.***

Dr. M. A. PALLEN, formerly of St. Louis, is now Professor of Gynæcology in the Med. Dep. of the Univ. of New York.

Surgeon JOHN FULLERTON BEATSON has been appointed Surgeon General of the Bengal Army.

Prof. W. F. PECK, a former graduate of Rush Med. College, is President of the Iowa State Med. Society.

GODFREY WALKER—also a graduate of Rush, who has since devoted especial and successful atention to ophthalmic and aural surgery, locates in Rochester, N. Y., for the future. He has the best wishes and highest expectations of all who know him.

Prof. ALFRED C. POST has resigned the chair of Surgery in the Univ. Med. College, N. Y., and Prof. JOHN T. DARBY, late of South Carolina, reigns in his stead.

Dr. EDWARD B. STEVENS, late of the *Cincinnati Lancet*, announces a new publication, the *Central New York Jour. of Medicine and Surgery.* It will of course prove a success, for Bro. Stevens "knows how it is himself" from successful experience.

Prof. HUXLEY has over three hundred and fifty students in Edinburgh University.

MADAME BRES, who recently obtained a degree from the Faculty of Medicine in Paris, has been appointed the medical attendant of the Sultan's harem at Constantinople. It is reported that she was offered a large bonus if she would confine her practice strictly to the seraglio, but declined to do so, preferring more general work about the city.

HORACE WELLS—the dentist who did not (but almost did) discover anæsthesia, is being remembered by a colossal bronze statue to be erected in the city of Hartford—and our alphabetical friend Morton, who did discover it, has not, as we are aware, even a first-class tombstone. Such is fame! Even Boston allows its anæsthesia monument to stand uninscribed save to the discovery. The discoverer ignored.

Dr. WM. K. BOWLING, long time Editor of the *Nashville Journal of Med. & Surgery*, and late President of the American Medical Association, has finally retired from the editorial tripod. Since the days of DAVID MEREDITH REESE, the medical press of this country has not known a combination of qualities better adapted to adorn the editorial position. Personally genial and benevolent, they were alike sensitive to the slightest affront to the profession of their choice, and woe to the poor wight who aroused their anger. Under their hands medical journalism was no dry-as-dust cataloguing of stale facts and assumed truths, but a very chart and chronicle of the time vivified by all surrounding practical influences. But we have no time to-day for parallels or contrasts. The best wishes of all his ancient contemporaries and modern co-workers will follow him wherever he goes.

Prof. DE LASKIE MILLER gains a few days needed rest just now at Long Branch. It is safe for him because he is no politician, neither do we believe he could be tempted from the chair he so successfully fills by any office in the gift of the "government" now located at L. B., L. I.

The *Am. Med. Weekly* wickedly attacks the profession and the language, by asserting that "twisted hemp cures felons." This is not a *Personal.*

Prof. GROSS believes in blood-letting and disbelieves in the duality of syphilis. Alas! is it so? Are the true things not new, and the new things not true?

A volunteer correspondent, at Ann Arbor, of the *Philadelphia Med. & Surg. Reporter*, communicates that Prof. A. SAGER, Dean of the medical faculty, has resigned the position on account of the homœopathic complications.

It is understood that although the Board of Health of Chicago is in imminent peril of dissolution under the new city organization—nevertheless, the efficient superintendent, Dr. B. C. MILLER, is to be retained. We earnestly hope this is true.

EDITOR'S NOTE. The following communication was received by a distinguished alienist, a few days since, and is given to the professional world on its intrinsic merits, *verbatim, literatim, et punctuatim.* H.

CINCINNATI O. 16. June '75.

Dear Sir I take the liberty to address a few idears by way of suggesteng in the treatment of insane persons. Let their rooms always be with *Sothern Exposier*, *ie*, on the Sun side of the building and let in the sun light freely on their persons so as to cause profuse persperation daily, when, the sun shines : In other words give them sun baths daily, which will *vitalize* the whole system, and give vigor as it does to plants and flowers. For diet use much fresh fish as they contain phosforos which is food for the brain: also brand bread as the brand is the nutritious part of wheat and contains phosphorus and will build up the brain &c. *Always have persons sleap with the head* to the North—then the electricity will runn down the nervous system. The three suggestions I have practiced for years on myself, and on others with signal success. They may not be anything new to yourself, so I give them for what they are worth.

All agree that the Vitalizing practice for the cure of all diseases is the most successful and permanant. I think more of sun-baths, fish and brand as diet not only in insane cases, but for constant practice in families for the prevention and cure of diseases than all other modes of treatment put togather, with sleaping with the head to the North.

